# Vascular Effects of the Fetal Hemoglobin Inducer Agent 3-(1,3-Dioxoisoindolin-2-yl) Benzyl Nitrate

**DOI:** 10.3390/ph15111311

**Published:** 2022-10-24

**Authors:** Barbara Terroni, Luis Henrique Oliveira de Moraes, Aline Renata Pavan, Gerson Jhonatan Rodrigues, Jean Leandro Dos Santos

**Affiliations:** 1 School of Pharmaceutical Sciences, São Paulo State University (UNESP), Araraquara 14800-903, Brazil; 2 Department of Physiological Sciences, Federal University of São Carlos (UFSCar), São Carlos 13565-905, Brazil; 3 Institute of Chemistry, São Paulo State University (UNESP), Araraquara 14800-060, Brazil

**Keywords:** nitric oxide, vascular reactivity, endothelial dysfunction, nitric oxide donors

## Abstract

Vascular endothelium is a protective layer of cells lining the lumen of blood vessels that plays important roles by releasing factors responsible for controlling the vascular tone, regulating the expression of pro-inflammatory cytokines, and expressing adhesion molecules involved in vascular hemostasis. Imbalance of vascular properties leads to endothelial dysfunction (ED) and cardiovascular damage. Some diseases, such as sickle cell anemia, are characterized by ED with reduction in the levels of nitric oxide (NO). Previously, we have shown that the fetal hemoglobin inducer agent 3-(1,3-dioxoisoindolin-2-yl) benzyl nitrate (**Lapdesf-4c**) could act as NO donor, inhibiting platelet aggregation and reducing the inflammation associated with SCA. However, the vascular effect of this compound was not yet studied. Herein, we evaluated the effects of **Lapdesf-4c** in vascular reactivity experiments using aortic rings from male Wistar rats (300 g/90 days). We have found that **Lapdesf-4c** induced vasodilation in the presence (E+) or absence of endothelium (E−) with an average of EMax values of 101.8 ± 3.33% and 111.8 ± 3.21%. The mechanism of action was studied using 1H-[1,2,4]oxadiazolo[4,3-alpha]quinoxalin-1-one (ODQ), *L*-N^G^-nitroarginine methyl ester (*L*-NAME), and hydroxocobalamin. The EMax values for those pathways were hydroxocobalamin (30.6 ± 2.21%), ODQ (4.75 ± 0.51%), and *L*-NAME (109 ± 3.65), suggesting that **Lapdesf-4c** exhibits NO-dependent mechanisms. **Lapdesf-4c** was able to prevent angiotensin-induced ED after incubation of aorta rings for 1 h. We found based on the concentration–effect curve using acetylcholine (ACh) that pEC50 values for the control, Ang II, and combination of (Ang II + **Lapdesf-4c**) were 6.73, 6.46, and 7.15, respectively. In conclusion, **Lapdesf-4c** has emerged as a new drug candidate that can promote vasodilation and act as a protective agent against ED, being useful to prevent vascular damage.

## 1. Introduction

The vascular endothelium (VE) is characterized by an endothelial cells monolayer, covering the blood vessel’s lumen, which acts through paracrine, endocrine, and autocrine effects. VE modulates the vascular tone and regulates the expression of adhesion molecules (CAMs), interfering in the coagulation cascade and blood flow. Those intrinsic properties are correlated with the mediators produced by VE, which include nitric oxide (NO). NO has an important role in the control of vascular tone, promoting vascular relaxation and vasodilation, and maintaining vascular hemostasis [1,2]. In addition, it acts by modulating pathways, pro-inflammatory cytokines (IL-1β, IL-6, and IL-8), and platelet-activating factor (PAF). These modulations occur through strictly controlled interactions between endothelial cells and other cellular components, such as blood cells, the immune system, and proteins present in the blood [3].

The imbalances in the NO levels are prone to induce endothelial dysfunction (ED). Such a deleterious event is responsible for creating an environment in which pro-inflammatory and pro-coagulant cytokines are released, expression of adhesion molecules on the cell surface increases (i.e., E-selectin, ICAM-1, and VCAM-1), and vasoconstriction increases, thus, favoring the formation of clots [2].

ED could also be induced by the high expression of oxidases, especially xanthine oxidase and NADPH oxidase complex [4], both enzymes that participate in the tissue ischemia–reperfusion cascade. The activation of oxidases can contribute to increasing the reactive oxygen species (ROS) levels, aggravating vascular endothelial damage and hampering the activity of enzymes with antioxidant effects, such as the superoxide dismutase enzyme (SOD) [5]. Moreover, it is described that such imbalances are prone to increase levels of vasoconstrictor mediators, such as Angiotensin II (Ang II) and Endothelin-1 (ET-1). Due to the effect of such vasoconstrictors, a more pronounced reduction in the levels of NO and antioxidants is found, aggravating the ED [6,7].

**Figure 1:**
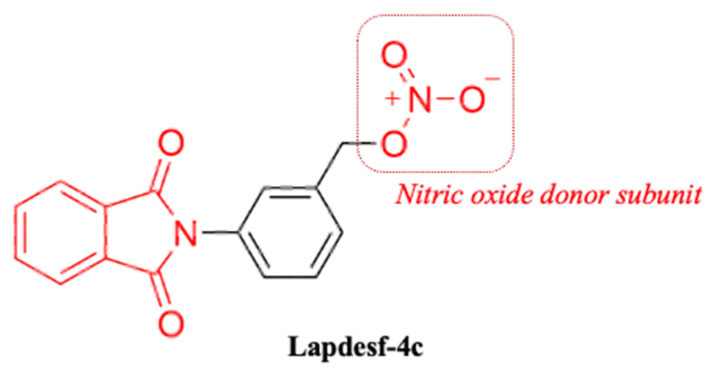
Chemical structure of 3-(1,3-dioxoisoindolin-2-yl) benzyl nitrate (**Lapdesf-4c**).

Compound 3-(1,3-dioxoisoindolin-2-yl) benzyl nitrate (**Lapdesf-4c**) (Figure 1) is described as the fetal hemoglobin-inducing agent that acts as NO donor, exhibiting pleiotropic effects as an anti-inflammatory and analgesic. These effects were associated, in parts, with the inhibition of pro-inflammatory cytokines, such as tumor necrosis factor-alpha (TNF-α) and IL-1 β [8]. In vitro and in vivo experiments showed that **Lapdesf-4c** did not exhibit mutagenic and genotoxic effects [9]. **Lapdesf-4c** also reversed the priapism in sickle transgenic murine models displaying low NO bioavailability after 3-week treatment by inducing phosphodiesterase-5 (PDE-5) expression and reducing protein expression of ROS markers [8,10]. Although such activities suggested a protective profile of **Lapdesf-4c** in the ED, there is no investigation of those vascular effects. Therefore, this work aims to evaluate the vasodilator potential of the compound and the endothelium participation in this response, in addition to verifying whether the compound is effective in promoting vasoprotection and if the antioxidant mechanism can reduce the ROS from the release of Ang II.

## 2. Results and Discussion

### 2.1. Vascular Reactivity Studies

**Figure 2:**
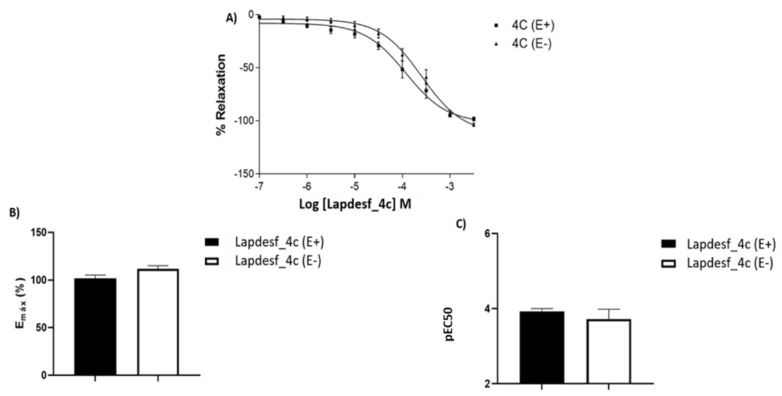
(**A**) Concentration–effect curves after treatment with **Lapdesf-4c** in the absence (E−) and presence of the endothelium (E+). (**B**) shows the Emax values expressed in percentage for **Lapdesf-4c** in the absence (E−) and presence of aortic intact endothelium (E+). (**C**) expresses the pEC50 values in the absence (E−) and presence of the endothelium (E+). Each experiment was performed on rings prepared from different rats.

Figure 2A shows the concentration–effect curve for **Lapdesf-4c** in rats’ aortic rings. It was found that this compound induced vasodilation in aortic rings through a non-endothelium-dependent manner (Figure 2A), since the relaxations were not different in the presence (E+) and absence (E−) of endothelium. The Emax values found were E+: 101.8 ± 3.3% (*n* = 9) and E−: 111.8 ± 3.2% (*n* = 7). In addition, the pEC50 values were E+: 3.9 ± 0.07% (*n* = 9) and pEC50 E−: 3.7 ± 0.26% (*n* = 7), revealing no differences between the groups.

**Figure 3:**
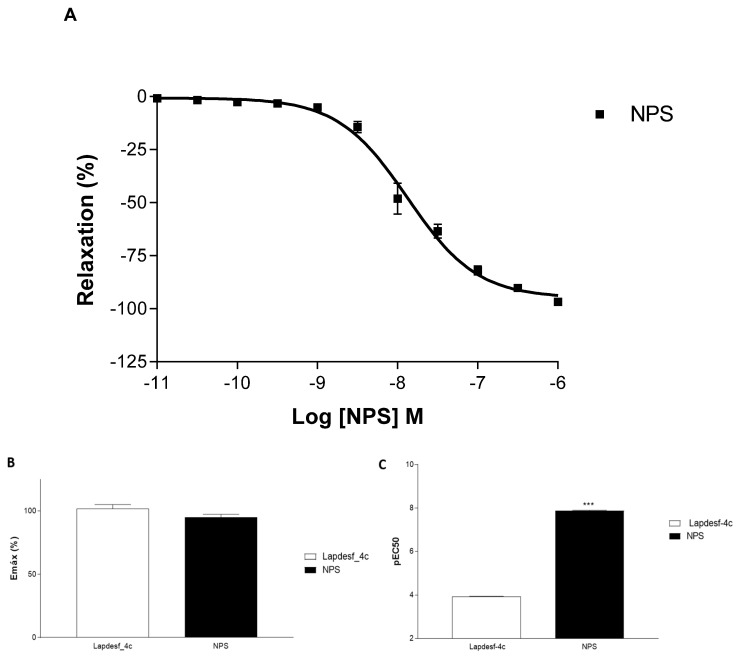
(**A**) Concentration–effect curves sodium nitroprusside (NPS) in the presence of the endothelium (E+). (**B**) shows the Emax values expressed in percentage for **Lapdesf-4c** (E+) and NPS (E+) without differences. (**C**) expresses the pEC50 values in the presence of the endothelium (E+) **Lapdesf-4c** and NPS *** *p* < 0.0001. Each experiment was performed on rings prepared from different rats.

As a parameter of efficacy and potency, sodium nitroprusside (NPS) was used as control (Figure 3). NPS is used in clinics as vasodilator, and the NO-release mechanism was already established. From its concentration–effect curve, it is possible to demonstrate that compound **Lapdesf-4c** (E+): 101.80; ±3.33%, (*n* =9) promotes vasodilation, as well as NPS ((E+): 94.99 ± 2.46%, (*n* = 8)). However, **Lapdesf-4c** does not exhibit the same potency ((E+): 3.92 ± 0.07%, (*n* =9)) as NPS ((E+: 7.87 ± 0.06% (*n* = 8)) (*p* < 0.0001).

### 2.2. Study of Vasodilation Mechanisms

Three different pathways were investigated to evaluate which mechanisms were involved in the vasodilation induced by **Lapdesf-4c**. For this purpose, (N*^G^*-mono-methyl-*L*-arginine ester) (*L*-NAME) (5 μM) was used as a non-selective NOS inhibitor, (1H)-[1,2,4]-oxadiazol(4,3-a)-quinoxaline-1-one (ODQ) (10 μM) was used as a soluble guanylate cyclase (sGC) inhibitor, and hydroxocobalamin (5 μM) was used as NO-scavenger.

**Figure 4:**
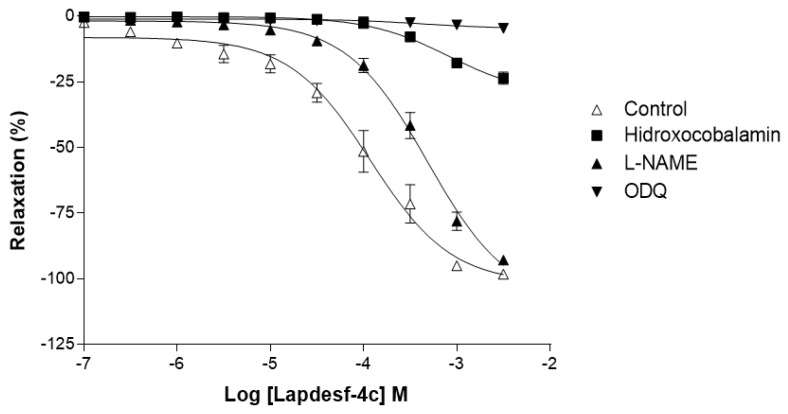
Concentration–effect curve for **Lapdesf-4c** (E+) in the presence of hydroxocobalamin, *L*-NAME, and ODQ to study the involvement of NO release pathway in vasodilation. Data are expressed as percentage of aortic relaxation. Each experiment was performed on rings prepared from different rats.

Figure 4 shows the percentage of relaxation induced by **Lapdesf-4c** (E+) in the presence of ODQ, hydroxocobalamin, and *L*-NAME. For ODQ, the relaxation induced by **Lapdesf-4c** (E+) was 4.79 ± 3.36% (*n* = 10); for hydroxocobalamin, it was 30.26 ± 3.87% (*n* = 9); and for *L*-NAME, it was 109 ± 3.31% (*n* = 9). In this experiment, the value for control was 101.8 ± 3.92% (*n* = 9).

### 2.3. Prevention of Endotelial Dysfuntion

**Figure 5:**
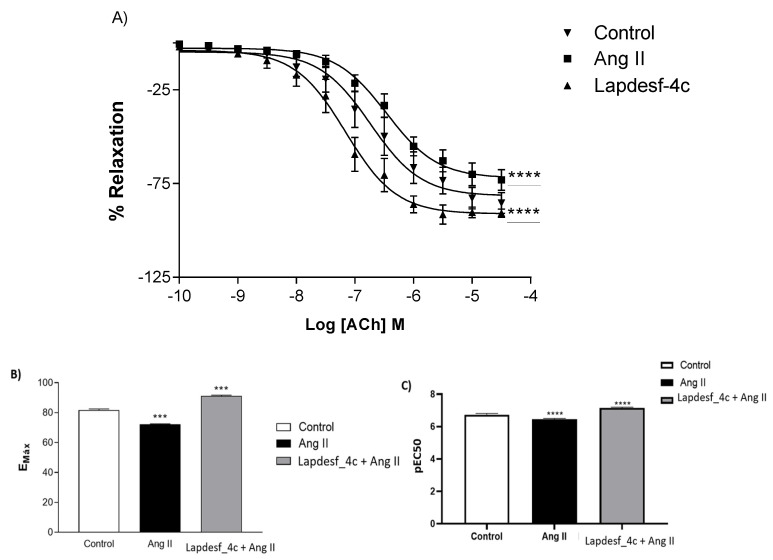
(**A**) Concentration–effect curve for acetylcholine (ACh) in the presence of endothelium (E+). The rings were incubated with Ang II. *** stands for *p* < 0.0001, which indicates difference between all groups. (**B**) EMax values obtained from the concentration–effect curve of the ACh. **** *p* < 0.0001 indicates difference between all groups, (**C**) pEC50 values obtained from the concentration–effect curve of the ACh. **** *p* < 0.0001 indicates difference between all groups.

The protective effect of **Lapdesf-4c** to prevent endothelial dysfunction (ED) induced by angiotensin II (Ang II) was studied. Previously, it was demonstrated that aortic rings pre-incubated with Ang II developed ED, measured through the ACh concentration–effect curve [11]. To determine whether **Lapdesf-4c** could prevent endothelial dysfunctions, one group was incubated with **Lapdesf-4c** (1 μM) for 30 min and then with Ang II (2 μM) for six hours. While the control group received only Krebs solution and the last group received only Ang II (2 μM), both stayed in the incubator for the same period. Our results demonstrate that this protocol induced ED, since the group with Ang II exhibited a percentage EMax value E+ of 72.11 ± 1.31% (*n* = 9) for ACh, while for the control group, the Emax value E+ was 81.60 ± 2.18% (*n* = 7). For **Lapdesf-4c**-treated groups, the values were EMax E+: 91.17 ± 1.45% (*n* = 7) (**Lapdesf-4c** + Ang II), showing a difference compared to control and treatment *p* < 0.0001 (Figure 4A,B). Figure 5C shows the difference in the potency expressed as pEC50. The treated group showed high pEC50 values (7.15 ± 0.1% (*n* = 7)) in comparison with the other two groups control (6.7 ± 0.1% (*n* = 7)) and Ang II (6.5 ± 0.1% (*n* = 9)) *p* < 0.0001. Based on these results, we concluded that **Lapdesf-4c** exhibits a protective effect in the Ang II-induced endothelial dysfunction experiments.

### 2.4. Intracellular NO Measurement

The indirect measurement of the intracellular levels of NO was carried out through incubation of HUVEC-treated cells with **Lapdesf-4c** in the presence and absence of Ang II using 4,5-diaminofluorescein (DAF-2DA), as fluorescent probe, at 10 μM. The objective of this experiment was to demonstrate for **Lapdesf-4c** at 1 μM, in which NO should be released, the abolishment of hypothetical bias in the concentration–effect curve of Ach.

**Figure 6:**
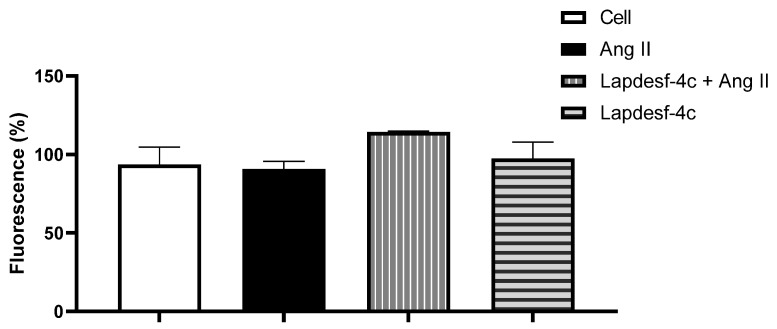
Percentage of fluorescence using DAF-2DA as probe after incubation of HUVEC cells with Ang II, **Lapdesf-4c**, and combined treatment (**Lapdesf-4c** + Ang II) without differences.

In aqueous medium, NO is converted through oxidative reactions into dinitrogen trioxide (N_2_O_3_). Thus, the fluorescence product obtained after the reaction of N_2_O_3_ with DAF-2DA is detected by fluorometer at 435 nm excitation and 538 nm emission wave [11]. Figure 6 shows the percentage of fluorescence for different groups. For the control group containing only HUVEC cells, the average percentage of fluorescence was 93.6 ± 11% (*n* = 3). The Ang II-treated group exhibited an average value of 90.6 ± 4.8% (*n* = 3). For **Lapdesf-4c**-treated group, the percentage found was 97.4 ± 10% (*n* = 3), while for a combination of **Lapdesf-4c** + Ang II, this value was 114.3 ± 0.6% (*n* = 3), showing no differences among them.

### 2.5. Measurement of Superoxide Anion Production

**Figure 7:**
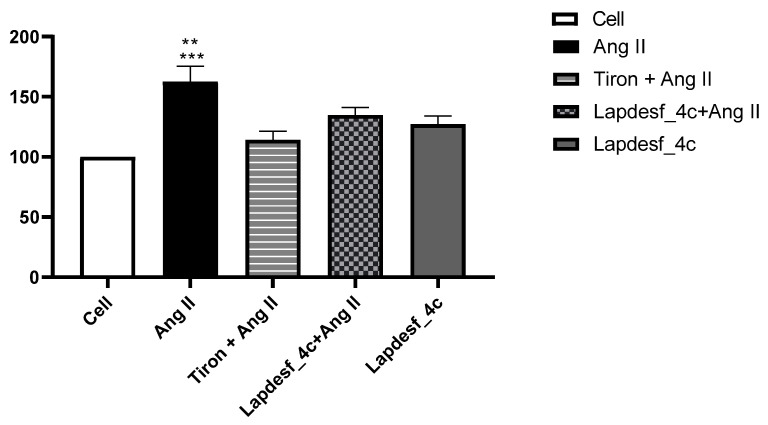
Percentage of fluorescence using lucigenin as probe after incubation of HUVEC cells with Ang II, **Lapdesf-4c**, and combined treatment (**Lapdesf-4c** + Ang II) and (Tiron + Ang II). O_2_^−^ detection, where ** *p* < 0.005 Ang II vs. 4c and 4c + Ang II. *** *p* < 0.001 Ang II vs. Tiron + Ang II.

The measurement of superoxide anion production was carried out to investigate whether the **Lapdesf-4c** (1 μM) endothelial protection was due to a reduction in the levels of O_2_^−^. Therefore, we investigated whether such reduction correlated with its vasoprotective effect demonstrated in the experiment, Section 2.3. In this experiment, the following groups were used: control (cell), Ang II, Tiron + Ang II, **Lapdesf-4c**, and **Lapdesf-4c** + Ang II. Tiron was used as control, due to its antioxidant effect (Figure 7).

Figure 7 shows the percentage of fluorescence for detection of O_2_^−^ in the cell group (100 ± 0% (*n* = 3)), Ang II (165 ± 12% (*n* = 3)), Tiron (114 ± 6% (*n* = 3)), **Lapdesf-4c** (127 ± 6% (*n* = 3)), and Ang II + **Lapdesf-4c** (134 ± 6% (*n* = 3)). Ang II induces superoxide production in endothelial cells, when compared with the cell group (*p* < 0.0001) and Ang II + **Lapdesf-4c** (*p* < 0.001). **Lapdesf-4c** -treated group decreased the superoxide formation induced by Ang II. A similar effect was observed for Tiron + Ang II group. Altogether, these results show that **Lapdesf-4c** reduces the superoxide anion production induced by Ang II.

### 2.6. Cellular Viability Assay

**Figure 8:**
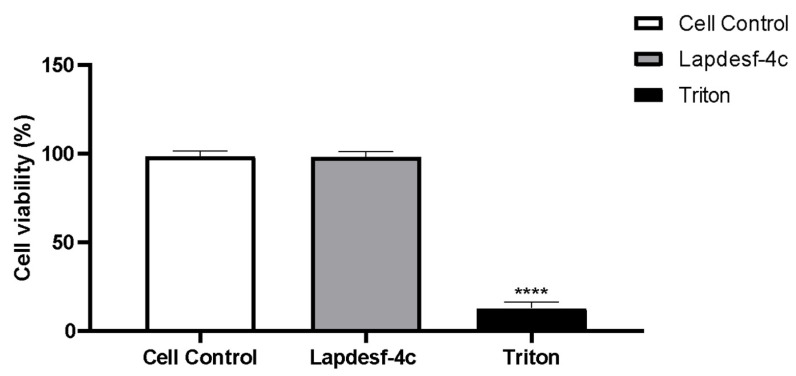
Cellular viability of HUVEC cells treated with **Lapdesf-4c** (200 μM) and triton (1 μM). **** *p* < 0.0001 compared to control and **Lapdesf-4c**.

Cellular viability in the presence of **Lapdesf-4c** (200 μM) was evaluated using HUVEC cells. In this experiment, the highest concentration used in the vascular reactivity concentration-effect curve was chosen for this experiment. Figure 8 shows that for the **Lapdesf-4c** group, the cellular viability was 98.3 ± 3% (*n* = 3), similar to that found for the cell control group (98.6 ± 2.8% (*n* = 3)). For the group treated with Triton (death control) at 1 μM, the cell viability was 12.9 ± 3.3% (*n* = 3). These results suggest that up to 200 μM, **Lapdesf-4c** did not demonstrate cytotoxic effect compared to Triton.

## 3. Discussion

ED is characterized by low levels of NO, increased expression of adhesion molecules (i.e., VCAM-1, ICAM-1), endothelin-1, Ang II, and pro-inflammatory cytokines (i.e., TNF-α, IL-1β, and IL-6). These deleterious environmental factors are prone to induce hypertension, clot formation, and vaso-occlusion [11,12,13]. It has been described that NO donors can attenuate hypoxia, decrease cell adhesion, and release pro-inflammatory cytokines. Moreover, NO donors stimulate soluble guanylate cyclase (sGC) and decrease leukocyte adhesion in the vascular lumen [14].

Strategies aiming to maintain appropriate levels of NO are important as therapeutic interventions to decrease the worsening of ED disease [12,14] Considering that **Lapdesf-4c** acts as NO donor and is efficient to promote endothelium-independent vasodilation, it should be considered as a promising agent to prevent ED.

An interesting study to characterize the vasodilation in ED in humans through endothelium-dependent or independent mechanisms compared treatments after an intra-arterial infusion of NPS (a NO donor), *L*-NAME (non-selective eNOS blocker), and methacholine (ACh analog). Brachial blood flow was monitored to assess the degree of arterial vasodilation [15]. It was found that ED patients obtained a better response than healthy patients for NPS. Furthermore, the inhibition of eNOS by *L*-NAME induced an inferior response in ED patients for methacholine, compared to healthy patients. Thus, these data demonstrated a reduced participation of endogenous NO in endothelium-dependent vasodilation. ED patients demonstrated lower levels of endogenous NO and a good response to exogenous NO donors [15].

In this work, the NO donor **Lapdesf-4c** was evaluated using the aorta artery of rats to study its potential vasodilator effect in the presence (E+) or absence (E−) of the endothelium. The results show that the percentage of vasodilation for **Lapdesf-4c** in the presence (E+) and absence (E−) of the endothelium were similar, not showing any differences between them, suggesting that **Lapdesf-4c** promotes endothelium-independent vasorelaxation (Figure 2A). NPS, a well-established NO donor used in clinics, was used as a control in our experiments. Both **Lapdesf-4c** and NPS act as sGC stimulators. However, pEC50 e Emax values for NPS are superior to those values found for **Lapdesf-4c** (Figure 3).

To study the mechanisms by which **Lapdesf-4c** acts, vasodilation studies were carried out in the presence of hydroxocobalamin (NO scavenger), ODQ (sGC inhibitor), and *L*-NAME (eNOS inhibitor). Figure 4 shows that *L*-NAME did not affect the maximum percentage of vasodilation, although it reduced the potency of the drug, according to its pEC50 values. The attenuated effect is due to the influence of endothelium on vascular tonus, which acted independently of the vasodilator mediator. Nitric oxide induces its synthesis, through the activation of GCs and accumulation of cGMP in endothelial cells [16]. Our data corroborated the study of vasodilation in the presence and absence of endothelium and demonstrated that **Lapdesf-4c** was effective to promote vasodilation in the absence of endothelium. However, ODQ and hydroxocobalamin were effective to inhibit vasodilation induced by **Lapdesf-4c**, suggesting the involvement of NO pathway in our observations (Figure 4).

For ODQ, it was reported that some NO donors can produce a vasodilator effect even in the presence of sGC inhibitors, such as linsidomine. However, other NO donors are unable to produce such an effect, due to the reflex blockage that exists to cGMP. The GCs blockage was already described for NPS [17]. In our results, we conclude that **Lapdesf-4c** promotes vasodilation independently of the endothelium; however, it is necessary to activate tissue through the cGMP pathway to promote vasodilation.

Vasculopathy in ED has been correlated with the low bioavailability of NO and increased levels of vasoconstrictors, such as Ang II and ET-1 [6,18]. Studies demonstrated that the interaction of Ang II with AT1 receptors in endothelial cells and vascular smooth muscle induces AT1 to activate the production of bET-1, a precursor to ET-1, which induces vasoconstriction and vascular/myocardial hypertrophies. Therefore, high levels of ET-1 are considered a cardiovascular risk for cardiovascular diseases [19]. Furthermore, Ang II is correlated with an overstimulation of the sympathetic nervous system (SNS). Thus, ROS and ET-1 released by Ang II can stimulate the SNS, increasing its constricting response in the vasculature and developing an increase in sympathetic activity, which in turn is associated with the worsening of ED [20]. Assuming that ED patients have high levels of Ang II, ROS, ET-1, and chronic activation of the SNS, it is reasonable that such patients are more prone to develop ED [7,20,21]. In this context, compounds acting to protect against ED are useful to prevent cardiovascular damage. Previous work conducted by Sanchez and colleagues (2007) demonstrated that the incubation of aortic rings with Ang II (1 µM) induces ED in the vessels [13]. Based on the work of Sanchez, the present study obtained similar data to those of Sanchez. After incubation of the rings with Ang II, a decrease in the response of the concentration–effect curve of Ach was observed, characterizing the ED. However, the results show that the previous incubation with **Lapdesf-4c** promoted endothelial protection by comparasion of the Emax values for both groups, suggesting the vasoprotective effect of **Lapdesf-4c** (*p* < 0.0001) (Figure 5).

We evaluated the protective effect of **Lapdesf-4c** to prevent ED. For this, based on the concentration–effect curve, a low concentration of **Lapdesf-4c** was used in order to avoid interfering with the Ach response, as demonstrated by the DAF-2DA experiment (Figure 6). Indirect measurement of intracellular NO levels showed no differences for increased intracellular NO levels at the concentrations tested.

Previous reports have described that treatment with S-nitrous acetyl DL-penicillamine (SNAP), another NO donor, was able to cause gene suppression of AT1-type receptors. In addition to vasodilation properties, NO donors downregulate AT1 levels, which could be useful to prevent ED [22]. Another work demonstrated that exposure of endothelial cells to different NO donors increased the gene expression of the Ang II receptor (AT2), which has opposite effects to AT1 [22]. High levels of Ang II increase the number of reactive oxygen species through the activation of the NADPH oxidase complex [6]. Ang II binding to the AT1 receptor occurs through the activation of G protein, promoting gp91phox activation. This intracellular mediator is regulated by the p47phox subunit, which undergoes phosphorylation and activates the Nox-1 subunit of the NADPH oxidase complex. Thus, it affects the catalytic activities and releasing of ROS, especially O_2_^−^, which reacts with NO to form NOOO^−^. High levels of NOOO^−^ induce vessel inflammation, release pro-inflammatory cytokines, and induce the expression of adhesion molecules. Altogether, these events are prone to cause more endothelial damage [18,20]. ROS are potent secondary messengers that mediate signaling in pathways, whose increased levels favor vessel inflammation and change the oxidative state of the iron present in the heme groups. The interactions between Ang II signaling and ROS lead to changes in the structural and functional characteristics of the vasculature [23].

Silva and colleagues (2016) demonstrated that **Lapdesf-4c** reduces the expression of gp91phox, responsible for increasing the NADPH oxidase complex [10]. Based on these previous results and the effect of the compound in promoting endothelial protection, the present study decided to verify if, at the concentration used (1 μM), there was a decrease in O_2_^−^. According to the results (Figure 7), it was found that **Lapdesf-4c** was able to promote the reduction of O_2_^−^ levels. The lucigenin experiment was also used to observe whether **Lapdesf-4c** was able to reduce the levels of O_2_^−^, at concentrations at which it did not release NO. Our results corroborate the previous observation from Silva and colleagues, since **Lapdesf-4c** reduced the levels of O_2_^−^ in HUVEC cells, exhibiting cardiovascular protection effects. **Lapdesf-4c** reduced the percentage of fluorescence levels compared to the Ang II group (*p* < 0.001), suggesting that this compound exhibits an antioxidant effect, since in both experiments, we used concentrations that do not release NO. Silva et al. (2016) demonstrated in their previous studies on the compound that it does not present cytotoxicity (300 μM), which is in accordance with our results [8,9]. **Lapdesf-4c** is not cytotoxic for HUVEC cells up to high concentrations (200 μM).

## 4. Materials and Methods

Male Wistar rats, weighing 280–300 g, were housed at the Federal University of São Carlos (UFSCar) according to institutional guidelines for good laboratory practices using animals. Rats were maintained in temperature-controlled facilities on a 12 h light–dark cycle with ad libitum food (standard rat chow) and water access. All the procedures were previously approved by the Animal Care and Use Committee of the UFSCar (Protocol number CEUA nº 1295101219).

### 4.1. Vascular Reactivity Studies

The rats were euthanized by decapitation, the thoracic aortas were isolated, and *n* = 10 were used, with each aortic ring coming from the aorta of a different rat. Aortic rings were cut in 4 mm length, placed in bath chambers for isolated organs containing Krebs solution (5 mL) at 37 °C, and continuously bubbled with 95% O_2_ and 5% CO_2_, pH 7.4, in an isometric myograph (Mulvany-Halpern-model 610 DMT-USA, Marietta, GA, USA). The data were recorded by a PowerLab8/SP data acquisition system (AD Instruments Pty Ltd., Colorado Springs, CO, USA).

The aortic rings were submitted to a tension of 1.5 g, which was readjusted every 15 min throughout a 60 min equilibration period before the addition of the tested drug. Endothelial integrity (E+) was assessed by the degree of relaxation induced by 1 μM acetylcholine, after contraction of the aortic ring by phenylephrine (0.1 μM). The ring was discarded if relaxation with acetylcholine was lower than 80%. After the endothelial integrity test, aortic rings were pre-contracted with phenylephrine (0.1 μM), and then concentration–effect curves for **Lapdesf-4c** were obtained. The curves were also obtained in the absence of endothelium (E−), which was mechanically removed by rolling the lumen of the vessel on a thin wire. Endothelial integrity was assessed by relaxation induced by 1 μM of acetylcholine after contraction of the aortic ring by phenylephrine (0.1 μM). Sodium nitroprusside was used as control (NPS) (0.01 µM to 100 µM). The maximum relaxant effect (EMax) and the potency values (pEC50) were obtained. Each experiment was performed on rings prepared from different rats.

### 4.2. Mechanisms Pathways

Aortic rings with endothelium (E+) were treated for 30 min using different inhibitors, as described: (NG-mono-methyl-L-arginine ester) (L-NAME) at 5 μM, as a non-specific NOS inhibitor; ((1H)-(1,2,4)-oxadiazol(4,3-a)-quinoxaline-1-ona)) (ODQ) at 10 μM, as a soluble guanylate cyclase (sGC) inhibitor; hydroxocobalamin at 5 μmol/L, as NO scavenger. After incubation, aortic rings were pre-contracted using phenylephrine (0.1 μmol/L), and concentration–effect curves for **Lapdesf-4c** were obtained.

### 4.3. Prevention of Endothelial Dysfunction

Aortic rings were placed in 6 wells containing a Krebs solution (5 mL), antibiotic–antimycotic mixture (penicillin (50 µL) and amphotericin B (2.5 µL)) in a cell culture incubator in the absence or presence of the treatment with **Lapdesf-4c** (1 μM) for one hour and after with Angiotensin II (Ang II) (2 μM) for six hours. Then, aorta rings were immediately used for contractile tension, and the values were recorded as described in Section 2.2, providing the acetylcholine (ACh) curve. The Emax and the pEC50 were obtained and analyzed. Each experiment was performed on rings prepared from different rats.

### 4.4. Quantification of NO Levels

HUVEC cells (between the 15th and 20th passage) were placed in 96-well plates at the concentration of 10⁴ cells per well. The plate was incubated for 24 h in a humidified incubator containing 5% CO_2_, at 37 °C. After 24 h, the treatment with the Lapdesf-4c (1 μM) was carried out, and the plates were incubated for 30 min in a humidified incubator. After the treatment, the Lapdesf-4c (1 μM) was removed, and the plates were gently washed with Phosphate Buffer Saline (PBS). The detection of intracellular NO was performed by incubation with a selective fluorescent probe 4,5-diaminofluorescein (DAF-2DA at 10 μM) for 30 min, to react with dinitrogen trioxide (N_2_O_3_) (an oxidation product of NO) and produce the fluorescent compound DAF-2DA. The reading was carried out with a SpectraMax GeminiXS fluorometer (Molecular Devices) at 435 nm excitation and 538 nm emission wavelength pair [24]. 

### 4.5. Measurement of Superoxide Anion Production

HUVEC cells were seeded in 96-well plates at 104 cells per well and maintained at 37 °C in a humidified incubator containing 5% CO_2_ for 24 h. Cells were treated with **Lapdesf-4c** (1 μM) and lucigenin for 30 min, followed by treatment with Ang II 0.1 μmol/L for 30 min. The increase in fluorescence intensity was monitored using a fluorescence microplate reader (SpectraMaxGeminiXS, Molecular Devices) at 510 nm and 595 excitation wavelength pair.

### 4.6. Cellular Culture and Cell Viability Assay

Immortalized human umbilical endothelial cells (HUVEC) were grown in a DMEM medium (Inlab) supplemented with 10% of fetal calf serum, antibiotics, and antimycotics. Cultures were maintained at 37 ± 2 °C in a 5% CO_2_ atmosphere. The cells in confluence were trypsinized, centrifuged at 1200 rpm for 5 min, and plated in 96-well plates.

HUVEC cells were plated at a concentration of 5 × 10^4^ cells per well and maintained at 37 °C in a humidified incubator containing 5% CO_2_ per 24 h. Cells were treated with Lapdesf-4c (200 μM) and triton 1%. Then, the viability of the cells was measured by using a colorimetric assay with MTT ([3-(4,5-dimethylthiazol-2-yl)-2,5-diphenyltetrazolium bromide). After 24 h of incubation, the medium was displaced, and 200 μL of MTT (5 mg/mL) was incubated for 4 h to form formazan crystals. Then, this solution was replaced by 100 μL of dimethyl sulfoxide (DMSO 100%) in each well and was incubated for 5 min, and the absorbance was read at 550 nm.

### 4.7. Statistical Analysis

Statistical analysis of the results was performed using GraphPad Prism version 8.0. Statistical significance was tested by one-way ANOVA (post hoc test: Newman–Keuls). Data are expressed as mean ± DP. In each set of experiments, n indicates the number of animals used in the study. Values of *p* < 0.05 were considered significant.

## 5. Conclusions

Several factors trigger ED, including low levels of endothelial NO, increased levels of Ang II, endothelin-1, and pro-inflammatory cytokines. NO has an important role in ED, and NO donors have been described as useful to decrease damage induced by ED. Here, the drug candidate **Lapdesf-4c** was able to induce vasodilation in the aorta obtained from rats independently from the endothelium. Mechanistic studies revealed that the release of NO is involved in the mechanism of vasodilatation. **Lapdesf-4c** did not show cytotoxicity, and it reduced the levels of O_2_^−^ and prevented ED in the aorta of rats induced by Ang II. Therefore, all these data suggest that **Lapdesf-4c** could be useful in preventing vascular damage in ED.

## Data Availability

The data presented in this study are openly available in Repositório Institucional UNESP at reference number http://hdl.handle.net/11449/214889.

## References

[1] Hadi H.A.R., Carr C.S., Al Suwaidi J. (2005). Endothelial Dysfunction: Cardiovascular Risk Factors, Therapy, and Outcome. Vasc. Health Risk Manag..

[2] Incalza M.A., D’Oria R., Natalicchio A., Perrini S., Laviola L., Giorgino F. (2018). Oxidative Stress and Reactive Oxygen Species in Endothelial Dysfunction Associated with Cardiovascular and Metabolic Diseases. Vascul. Pharmacol..

[3] Kirkpatrick C.J., Wagner M., Hermanns I., Klein C.L., Köhler H., Otto M., van Kooten T.G., Bittinger F. (1997). Physiology and Cell Biology of the Endothelium: A Dynamic Interface for Cell Communication. Int. J. Microcirc. Exp..

[4] Kato G.J., Steinberg M.H., Gladwin M.T. (2017). Intravascular Hemolysis and the Pathophysiology of Sickle Cell Disease. J. Clin. Investig..

[5] Alayash A.I. (2018). Oxidative Pathways in the Sickle Cell and Beyond. Blood Cells Mol. Dis..

[6] Dos Santos A.F., Almeida C.B., Brugnerotto A.F., Roversi F.M., Pallis F.R., Franco-Penteado C.F., Lanaro C., Albuquerque D.M., Leonardo F.C., Costa F.F. (2014). Reduced Plasma Angiotensin II Levels Are Reversed by Hydroxyurea Treatment in Mice with Sickle Cell Disease. Life Sci..

[7] Ergul S., Brunson C.Y., Hutchinson J., Tawfik A., Kutlar A., Webb R.C., Ergu A. (2004). Vasoactive Factors in Sickle Cell Disease: In Vitro Evidence for Endothelin-1-Mediated Vasoconstriction. Am. J. Hematol..

[8] Lanaro C., Franco-Penteado C.F., Silva F.H., Fertrin K.Y., Dos Santos J.L., Wade M., Yerigenahally S., de Melo T.R., Chin C.M., Kutlar A. (2017). A Thalidomide-Hydroxyurea Hybrid Increases HbF Production in Sickle Cell Mice and Reduces the Release of Proinflammatory Cytokines in Cultured Monocyte. Exp. Hematol..

[9] dos Santos J.L., Lanaro C., Lima L.M., Gambero S., Franco-Penteado C.F., Alexandre-Moreira M.S., Wade M., Yerigenahally S., Kutlar A., Meiler S.E. (2011). Design, Synthesis, and Pharmacological Evaluation of Novel Hybrid Compounds To Treat Sickle Cell Disease Symptoms. J. Med. Chem..

[10] Silva F.H., Karakus S., Musicki B., Matsui H., Bivalacqua T.J., Dos Santos J.L., Costa F.F., Burnett A.L. (2016). Beneficial Effect of the Nitric Oxide Donor Compound 3-(1,3-Dioxoisoindolin-2-Yl) Benzyl Nitrate on Dysregulated Phosphodiesterase 5, NADPH Oxidase, and Nitrosative Stress in the Sickle Cell Mouse Penis: Implication for Priapism Treatment. J. Pharmacol. Exp. Ther..

[11] Dao V.T.-V., Medini S., Bisha M., Balz V., Suvorava T., Bas M., Kojda G. (2016). Nitric Oxide Up-Regulates Endothelial Expression of Angiotensin II Type 2 Receptors. Biochem. Pharmacol..

[12] Gewaltig M.T., Kojda G. (2002). Vasoprotection by Nitric Oxide: Mechanisms and Therapeutic Potential. Cardiovasc. Res..

[13] Sanchez M., Lodi F., Vera R., Villar I.C., Cogolludo A., Jimenez R., Moreno L., Romero M., Tamargo J., Perez-vizcaino F. (2007). Quercetin and Isorhamnetin Prevent Endothelial Dysfunction, Superoxide Production, and Overexpression of P47 Phox Induced by Angiotensin II in Rat Aorta 1. J. Nutr..

[14] Setty B., Stuart M. (1996). Vascular Cell Adhesion Molecule-1 Is Involved in Mediating Hypoxia-Induced Sickle Red Blood Cell Adherence to Endothelium: Potential Role in Sickle Cell Disease. Blood.

[15] Eberhardt R.T., McMahon L., Duffy S.J., Steinberg M.H., Perrine S.P., Loscalzo J., Coffman J.D., Vita J.A. (2003). Sickle Cell Anemia Is Associated with Reduced Nitric Oxide Bioactivity in Peripheral Conduit and Resistance Vessels. Am. J. Hematol..

[16] Martinelli A.M., Rodrigues C.N.S., Moraes T.F., Rodrigues G.J. (2018). In Endothelial Cells, the Activation or Stimulation of Soluble Guanylyl Cyclase Induces the Nitric Oxide Production by a Mechanism Dependent of Nitric Oxide Synthase Activation. J. Pharm. Pharm. Sci..

[17] Homer K.L., Fiore S.A., Wanstall J.C. (1999). Inhibition by 1 H-[1,2,4]Oxadiazolo[4,3-a]Quinoxalin-1-One (ODQ) of Responses to Nitric Oxide-Donors in Rat Pulmonary Artery: Influence of the Mechanism of Nitric Oxide Generation. J. Pharm. Pharmacol..

[18] Forrester S.J., Booz G.W., Sigmund C.D., Coffman T.M., Kawai T., Rizzo V., Scalia R., Eguchi S. (2018). Angiotensin II Signal Transduction: An Update on Mechanisms of Physiology and Pathophysiology. Physiol. Rev..

[19] Sudano I., Spieker L.E., Herman F., Flammer A., Corti R., Noll G., Luscher T.F. (2006). Protection of Endothelial Function: Targets for Nutritional and Pharmacological Interventions. J. Cardiovasc. Pharmacol..

[20] Majumder K., Wu J. (2015). Molecular Targets of Antihypertensive Peptides: Understanding the Mechanisms of Action Based on the Pathophysiology of Hypertension. Int. J. Mol. Sci..

[21] Morris C.R. (2008). Mechanisms of Vasculopathy in Sickle Cell Disease and Thalassemia. Hematology.

[22] Ichiki T., Usui M., Kato M., Funakoshi Y., Ito K., Egashira K., Takeshita A. (1998). Downregulation of Angiotensin II Type 1 Receptor Gene Transcription by Nitric Oxide. Hypertension.

[23] Meht P.K., Griendling K.K. (2007). Angiotensin II Cell Signaling: Physiological and Pathological Effects in the Cardiovascular System. Am. J. Physiol. Physiol..

[24] Nakatsubo N., Kojima H., Kikuchi K., Nagoshi H., Hirata Y., Maeda D., Imai Y., Irimura T., Nagano T. (1998). Direct Evidence of Nitric Oxide Production from Bovine Aortic Endothelial Cells Using New Fluorescence Indicators: Diaminofluoresceins. FEBS Lett..

